# Healthcare Worker Serious Safety Events: Applying Concepts from Patient Safety to Improve Healthcare Worker Safety

**DOI:** 10.1097/pq9.0000000000000434

**Published:** 2021-06-23

**Authors:** Christine Foster, Lauren Doud, Tua Palangyo, Matthew Wood, Rick Majzun, Jessey Bargmann-Losche, Lane F. Donnelly

**Affiliations:** From the * Environmental Health and Safety, Lucile Packard Children’s Hospital Stanford, Palo Alto, Calif.; † Center for Pediatric and Maternal Value, Lucile Packard Children’s Hospital Stanford, Palo Alto, Calif.; ‡ Administration, Lucile Packard Children’s Hospital Stanford, Palo Alto, Calif.; § Stanford University, School of Medicine, Palo Alto, Calif.

## Abstract

**Introduction:**

Patient safety has improved pediatric healthcare by defining when patient safety events meet criteria as serious safety events (SSEs). Similar concepts apply to healthcare worker (HCW) safety. We describe the newly designed process for HCW injury reporting, the process for evaluating HCW SSEs, and early experience with the new systems.

**Methods:**

The work to redesign our approach to HCW safety included 2 parts: (1) process flow mapping and redesigning the work for HCW injury reporting; and (2) creating a process to categorize HCW injuries and determine when such injuries rise to a HCW SSE level. We evaluated the mean time per month from HCW injury to reporting and compared those values during the postimplementation time. We also evaluated the team’s experience with the first 4 potential HCW SSEs.

**Results:**

By improving the process flow, the mean time to reporting decreased significantly from 28 days implementation time-period (September–October 2019) to 9 days during the postimplementation time-period (November 2019–May 2020) (*P* = 0.0002). Of the first 4 HCW events identified and reviewed as possible HCW SSE events, there were 2 defined as HCW SSE level 4, one defined as a precursor event, and one defined as a nonsafety event.

**Conclusion:**

Adapting infrastructure and definitions used previously to improve patient safety can improve HCW safety.

## INTRODUCTION

By adopting a common framework of classifying patient serious safety events (SSEs), children’s hospitals across the United States have both individually and collectively improved patient safety by reducing the frequency of patient SSEs.^1–10^ Creating a common mental model for SSEs has allowed for better shared-learning and action plan development to avoid such events from happening.^1–10^ That collaborative effort between children’s hospitals has used definitions and algorithms defined by Healthcare Performance Improvement (HPI).^11^ HPI defined an SSE as a deviation from best-practice care, causation, and significant patient harm or death.^3,5,11^

Although there is increasing attention in the healthcare industry around the importance of healthcare worker (HCW) safety, there has been little published regarding the use of similar frameworks to improve HCW safety and decrease HCW SSEs. Our organization has recently undergone efforts to improve HCW safety by improving the timeliness of our HCW injury reporting and improving the effectiveness of action plans created in response to HCW injuries to decrease the likelihood of similar event recurrence. We describe the newly designed process for HCW injury reporting, the process for evaluating events as potential HCW SSEs, and early experience with the new systems.

## METHODS

This project met the criteria for a quality improvement project, was not considered human subjects research, and did not require our Institutional Review Board’s approval. We carried out this project at a pediatric health system that includes quaternary services in pediatrics and obstetrics, primary and subspecialty pediatric ambulatory services, and association with a large university.

This project aimed to improve our HCW injury reporting timeliness and improve upon the ability, when HCW injuries did occur, to create action plans to prevent such occurrences in the future. The work to redesign our approach to HCW safety included 2 parts: (1) process flow mapping and redesigning the work for HCW injury reporting and (2) creating a process to categorize HCW injuries and determine when such injuries rise to a HCW SSE level.

### Process Flow Mapping and Redesigning HCW Injury Reporting and Management

Initially, we informally examined the existing injury reporting and investigation process by conducting interviews with key stakeholders and evaluating historical data. This effort revealed several underlying challenges that directly or indirectly contributed to ineffective follow-up of HCW injury events. Notification of HCW injuries was significantly delayed, with many injuries not being reported to department leadership and Environmental Health and Safety (EHS) until the receipt of the previous month’s incident rate data. Although our Occupational Health Serviced department and our workers’ compensation carrier may have been aware of these injuries, leadership might not have been aware of an injury until up to 7 weeks after the event. Also, a paper-based document was completed by the supervisor following the injury. That paper was then scanned and sent to Human Resources, where it would be logged in a database and then sent to EHS. This paper copy was only completed for 25%–50% of HCW injuries, and sometimes the wrong individuals would receive the form. There was no formal process or expectations around injury event follow-up. Injury trending data were difficult to utilize based on inaccuracy and delays in reporting.

**Fig. 1.**
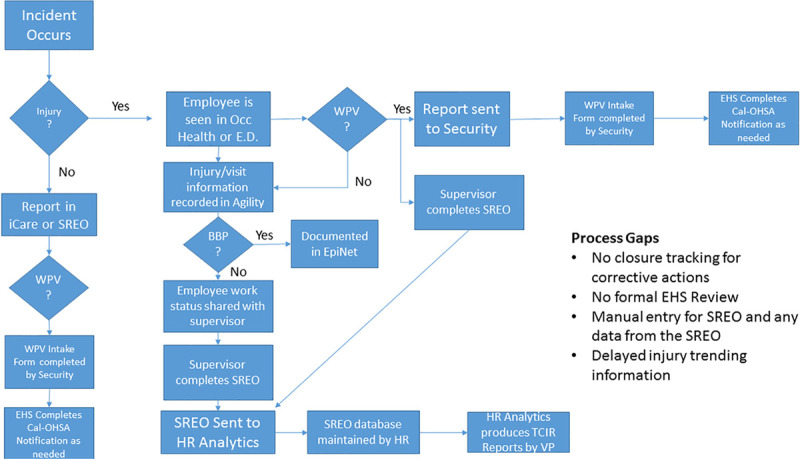
Process flow map showing the HCW injury process before redesign. BBP, blood-borne pathogens; EHS, environmental health and safety; EpiNet, this is a national software program that is used to records healthcare sharps injuries; SREO, Supervisor’s Report of Event or Occurrence (form used before we moved to iCare); TCIR, total case incident rate (number of recordable injuries per 100 employees); WPV, workplace violence.

The initial review then lead to process flow mapping of the entire reporting process from time of injury to notification (Fig. 1). This exercise revealed the complexity of the current system as well as the multiple potential points of failure. In addition to failure points within the existing reporting process, the form utilized for reporting the injuries did not capture essential information related to the event’s apparent or root cause. The form focused on capturing the event’s details, such as date, time, place, and individuals involved, and a brief description of the event. Although the form did include a question around executed preventative actions to prevent reoccurrence, supervisors did not understand how to effectively complete this question and often answered that the event was not preventable. Once supervisors completed that report, there were no additional expectations for follow-up on the event or a system approach to prevent the event from recurring.

**Fig. 2.**
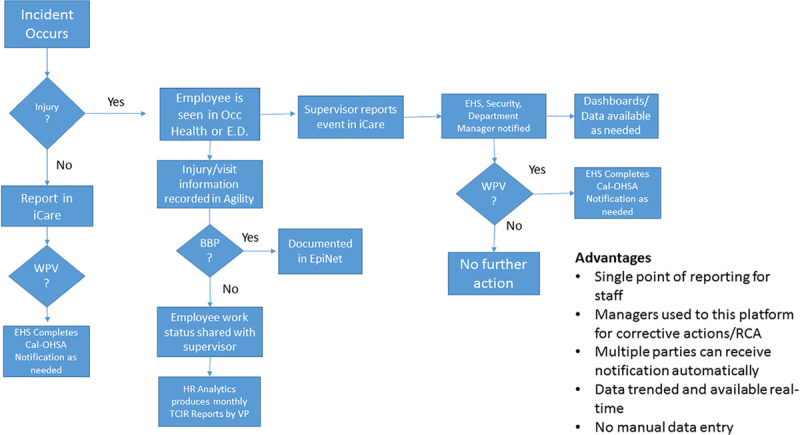
Process flow map showing the HCW injury process after redesign. BBP, blood-borne pathogens; EHS, environmental health and safety; EpiNet, this is a national software program that is used to records healthcare sharps injuries; SREO, Supervisor’s Report of Event or Occurrence (form used before we moved to iCare); TCIR, total case incident rate (number of recordable injuries per 100 employees); WPV, workplace violence.

The organization already had a robust patient safety-related incident reporting system internally configured and based on a commercially available product (RLDatix, Toronto, ON, Canada). With approximately 8,000 patient safety incident reports filed annually, there is a robust utilization of that system. Also, as part of the overall Culture of Safety, the organization strived to improve the prevention of HCW injuries. With that in mind, the decision was made to utilize the existing patient safety incident reporting platform for reporting and follow-up for HCW injury and near-miss events. As HCWs were already very familiar with the platform, they viewed it as more manageable for the team to go to one consistent place to report all safety-related events. New process flow was created based on the safety incident reporting system (Fig. 2) and a notification system that paralleled patient safety events. This notification included the manager of the employee, department director, and EHS for all events. For certain types of events, such as workplace violence, other parties were added to the automatic notification. The HCW injury report form mirrored the patient safety event-form to help make the HCW transition easier. The existing patient safety electronic form also included the elements missing from the previous paper-based form, including identifying the root cause, corrective actions, and reviewing those identified plans by a subject matter expert.

Table 1 summarized gaps identified in the baseline process and process improvements associated with the new system.

**Table 1.** Summary of Identified Gaps in Original Process and Process Improvement Solutions

Identified Gap in Original Process	Process Improvement Solution
Hard copy form was not completed consistently	All safety events reported through the same system, versus needing to utilize a separate system for HCW events
Hard copy form required manual routing to all stakeholders	Online patient safety system is built with automatic notification and routing
Hard copy form did not include robust prompts for helping identify causation and corrective actions	Patient safety forms already contained sections for identifying cause and corrective/follow-up actions
Hard copy form process did not include a closed loop review process to ensure completion of actions	Patient safety process included a final subject matter expert review to ensure that the follow-up actions were deemed adequate to address the issue and prevent reoccurrence
Hard copy process included up to 12 steps with 3 separate work flows, depending on type of injury. This created several points of possible failure	Patient safety electronic system has smart logic that helps route injury information to the correct stakeholders based on the type of injury. This reduced overall steps in the process to 4–5, with one standard workflow

### HCW SSE Classification

Our organization currently uses definitions and algorithms created by HPI to review and classify patient safety events as SSEs or precursor events.^11^ We also have a defined process by which all incident reports are reviewed and evaluated by safety staff, and any potential patient safety events identified through incident reports or other means are further investigated and reviewed at a weekly event review meeting and categorized promptly.^5^ As this infrastructure already existed, we elected to use that patient safety infrastructure to review potential HCW SSEs. We created HCW SSE criteria by adapting the HPI patient SSE classifications to HCW injuries (Table 2). Definition of a HCW SSE is a HCW injury that is significant per the created definition, occurred related to deviation from our standard practice, and caused significant harm. As our patient care SSEs, we categorize HCW SSEs as HCW SSEs 1–5 (Table 1). To define these 5 categories, we examined each of the existing Level of Harm definitions and determined a corresponding HCW injury level. For example, the definition of an SSE 2, Severe Permanent Harm, was “A deviation in generally accepted practice resulting in critical, life-changing harm with no expected changes in clinical status. This category includes events resulting in permanent loss of limb, organ, or vital physiologic or neurologic function.” The HCW example for this injury level was “Permanent physical disability from a work-related injury. Unable to return to work” (Table 1).

**Table 2.** HCW SSE Classification

Category	Harm Definition
HCW SSE 1 Death	Employee death
HCW SSE 2 Severe permanent harm	Permanent physical disability from a work related injury. Unable to return to work
HCW SSE 3 Moderate permanent harm	A work-related permanent disability that would prevent the employee from returning to the job they had previously held
HCW SSE 4 Severe temporary harm	Lost or restricted work time greater than 6 mo and/or emergency medical treatment needed
HCW SSE 5 Moderate temporary harm	Lost or restricted work time between 3 and 6 mo

Adapted with permission from Ref. ^11^.

Our EHS team now reviews all HCW injuries. If the HCW injury potentially meets the criteria of a HCW SSE and/or be an injury resulting from a system issue, the case is reviewed at our weekly event review meeting.^5^ Attendees of the weekly event review meeting include the Chief Operating Officer, Chief Medical Officer, Chief Quality Officer, Chief Nursing Executive, other essential nursing and physician leaders, patient safety leaders, and the Director of EHS. The Director of EHS presents the case summary. In addition to categorization, the group also determines whether an analysis and potential corrective action is needed. Potential actions include performing a root cause analysis or process review and creating a resulting action plan. Utilizing all of the existing platforms and processes for patient safety events would allow the organization to review all safety events, in the same way, ensuring that HCW events also received thorough follow-up and closure of identified corrective actions.

For events that do not meet the threshold as a potential HCW SSE, the area manager creates an action plan to help prevent that particular event from recurring. Mangers are expected to complete this task within 72 hours of the reported injury or illness event. The manager-created action plans are reviewed and approved by the Director of EHS.

In addition to the HCW SSE classification, all employee injuries are also classified as it pertains to Occupational Safety and Health Administration (OSHA) types of injuries and Occupational Safety and Health Administration recordable events, total case incident rate as well as *Days Away Restricted or Transferred*. The HCW SSE categorization is in addition to and not meant to replace these other forms of employee injury categorization.

### Evaluation

After moving to the new HCW injury and HCW SSE determination processes, we evaluated our initial experience with the new system. The implementation period was defined as the first 10 weeks, whereas the new process was rolled out. The postimplementation period was defined as the time from week 11 to week 47. We evaluated the mean time per week from HCW injury to reporting over time, comparing the time during the implementation to the postintervention period. We evaluated statistically significant differences in these times with a 2-sample T-test (R, R Core Team, Vienna, Austria). Data were displayed as a control chart with control limits calculated for each week based on implementation period data. We calculated control limits based on implementation period data. Based on these control limits, we looked for patterns in the postimplementation period that would permit us to invoke control chart rules that indicate a sustained decrease in the process mean. Our goal is to have the mean time per week from HCW injury to reporting to be less than 7 days.

We reviewed the team’s experience with the first 4 potential HCW SSEs, including any process reviews or root cause analysis that resulted from those reviews.

## RESULTS

### Timeliness of Reporting

During the implementation phase, there were a total of 54 reported HCW injuries. During the postimplementation phase, there were a total of 239 reported HCW injuries.

**Fig. 3.**
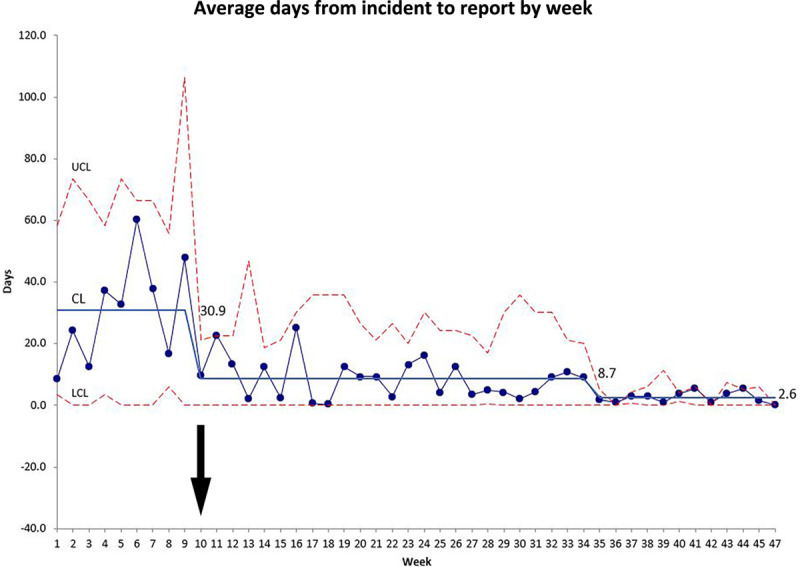
Control chart of reporting showing average time from employee injury to reporting averaged per week. Centerline indicates the straight blue lines. Dashed red lines indicates the control limits. Arrow indicates the beginning of the post implementation period.

The transition from a paper-based reporting form to the online safety event reporting system’s utilization decreased reporting time significantly. Although data on timeliness from the preimplementation period is not known, which was a problem in itself, time for an individual case could take as long as 7 weeks. The mean time to reporting decreased significantly from 28 days during the implementation period (September–October 2019) to 9 days during the postimplementation period (November 2019–May 2020) (*P* = 0.0002). Figure 3 shows data in a run chart. All weekly means in the postintervention period were lower than those in the intervention period justifying a change in the centerline. A second centerline change was justified after week 34. We now investigate all HCW injuries and create a corrective action plan for each. At this time, 16% of these online reports now have corrective actions documented within 72 hours of the event.

### Event Review and HCW SSE Determination

Of the 239 reported HCW injuries during the postimplementation period, to date, we have evaluated 4 events that were deemed to be potential HCW SSEs via the new event review process. Three of the 4 events involved latex exposure in the workplace, resulting in a need for immediate emergency medical attention. Two of the latex events involved exposure to hospital materials resulted in emergency room visits with treatment for anaphylaxis and were classified as SSE level 4. The event review group elected to perform a process review related to the management of latex in the organization based on the review of the cluster of the above events. A third case involving latex exposure was not deemed a HCW SSE as the exposure was not to hospital-acquired products and did not result in significant harm.

We performed a formal process review of the latex exposure events classified as SSE4s. The process review’s objective was to determine the current state of product purchasing and acquisition; identify gaps in that process related to the review of items for latex-containing materials, and create an action plan to address any identified process gaps.

A total of 12 action items were identified from those gaps, along with owners and due dates.

The fourth event reviewed involved a portable computed tomography scanner that had trapped a worker against a wall for a period of 5–7 seconds. The worker was unharmed. Due to the event’s outcome, the panel determined that this event was a precursor event. The event, however, did reveal a significant HCW system safety issue related to the faulty equipment. We placed the equipment out of service, reported it to the US Food and Drug Administration’s Medical Product Safety Network (FDA MedSun).

## DISCUSSION

We leveraged an existing patient safety platform to improve HCW injury reporting and the follow-up processes following an injury event. The underlying goal in improving reporting and follow-up is preventing future events and strengthening the safety culture. Studies have demonstrated that a positive safety culture reduces patient and HCW harm, increases worker satisfaction, and decreases turnover.^1–10,12,13,14^ Historically, in the healthcare space, the focus of safety programs has been on patient safety. HCW safety programs may not exist or may be sequestered from patient safety efforts. Examining industry data on nonfatal occupational injuries demonstrates that occupational injury and illness cases with HCWs are among the highest of any industry sector in the United States.^15^ A focus on HCW safety programs is clearly essential to ensure a successful overall hospital safety program. The majority of the literature to date on safety in healthcare has focused on patient safety.

Patient safety publications have demonstrated the value of harm assessment, score assignment, and consistent follow-up for reducing preventable harm. In industry publications outside of healthcare, these same concepts exist in the world of occupational safety. The concept of getting to zero is not unique to preventing patient harm, and best in class occupational safety programs apply these same principles to worker safety events.^16,17^

A singular focus on patient safety within a healthcare organization can erroneously send a message that HCW safety is not valued as highly as patient safety. Including HCW injury reporting and follow-up and HCW SSE event processes into an existing patient safety infrastructure integrates worker safety into the overall goal of zero preventable harm. In our work, we found that the redesign of the HCW injury reporting system to encompass all safety events within one central location provided benefits related to the efficiency of the process and sent a message from leadership that all safety events matter. Worker feedback on the process was very positive, with anecdotal comments from staff about how grateful they were to have someone reach out and follow-up on their injury. They also valued their ability to have a voice in the corrective action development. The HCW SSE review and follow-up process provide organizational visibility, structured problem solving, and leadership commitment. As is the case with the patient-facing event review process, the HCW SSE process also creates cross-disciplinary workgroups that help break down silos that may otherwise be barriers to solving HCW safety challenges. For example, the HCW SSE process reviews highlighted multiple gaps in the purchasing and acquisition process related to the safety review of items before purchase. They allowed key stakeholders and executive sponsors to tackle a complex problem that one department would not solve alone. In addition to reporting any HCW injuries daily through our tiered daily management system up to our executive huddle, there are many HCW injury metrics on our institutional key performance indicators dashboard that gets reported to our leadership monthly and to our governing board. These include the Days Away Restricted or Transferred rate and the percentage of employee injuries reported and investigated within 72 hours.

One of the limitations of this study is the amount of data collected to date. The HCW SSE process is new and thus has generated only the data presented here. We must collect additional data to determine the overall effectiveness of this integration. Additional research is also needed to determine how this change impacts the organization’s overall culture of safety.

In conclusion, we believe that adapting infrastructure and definitions shown to improve patient safety can improve HCW safety. It has led to improved efficiencies, such as decreased reporting times for HCW injuries and increased incident data quality. It sends a message to our workforce that HCW safety is essential and integral to patient safety.

## DISCLOSURE

The authors have no financial interest to declare in relation to the content of this article.
